# Multiple choice questions are superior to extended matching questions to identify medicine and biomedical sciences students who perform poorly

**DOI:** 10.1007/s40037-013-0068-x

**Published:** 2013-07-11

**Authors:** Thijs M. H. Eijsvogels, Tessa L. van den Brand, Maria T. E. Hopman

**Affiliations:** 1 Department of Physiology (392), Radboud University Nijmegen Medical Centre, PO Box 9101, 6500 HB Nijmegen, The Netherlands; 2 TriamFloat, Utrecht, The Netherlands

**Keywords:** Binding study advice, Medical education, Medicine, Biomedical sciences, Propaedeutic phase, Age

## Abstract

In recent years, medical faculties at Dutch universities have implemented a legally binding study advice to students of medicine and biomedical sciences during their propaedeutic phase. Appropriate examination is essential to discriminate between poor (grade <6), moderate (grade 6–8) and excellent (grade ≥8) students. Therefore, we compared the discriminatory properties of extended matching questions (EMQs) versus multiple-choice questions (MCQs) and identified the role of sex, age and examination preference on this score. Data were collected for 452 first-year medical and biomedical science students during three distinct course examinations: one examination with EMQ only, one with MCQ only and one mixed examination (including EMQ and MCQ). Logistic regression analysis revealed that MCQ examination was 3 times better in identifying poor students compared with EMQ (RR 3.0, CI 2.0–4.5), whereas EMQ better detected excellent students (average grade ≥8) (RR 1.93, CI 1.47–2.53). Mixed examination had comparable characteristics to MCQ. Sex and examination preference did not impact the score of the student. Students ≥20 years had a 4-fold higher risk ratio of obtaining a poor grade (<6) compared with students ≤18 years old (RR 4.1, CI 2.1–8.0). Given the strong discriminative capacity of MCQ examinations to identify poor students, we recommend the use of this type of examination during the propaedeutic phase of medicine and biomedical science study programmes, in the light of the binding study advice.

## Introduction

In recent years, medical faculties at Dutch universities have implemented a legally binding study advice that determines the minimum level of performance students must achieve during the propaedeutic phase of medicine and biomedical science study programmes. For the medical faculty of the Radboud University Nijmegen, a binding study advice entails that a first-year student has to earn at least 40–42 out of 60 credits (ECTS) to remain in the study programme [1]. To discriminate between eligible and ineligible students during the propaedeutic phase of medicine and biomedical science study programmes, appropriate examination is essential. In the propaedeutic phase of the study programmes for medicine and biomedical sciences, 4-week courses are assessed by a written examination including multiple-choice questions (MCQs), extended matching questions (EMQs), or a combination of the two [2].

Traditional MCQs require students to select the best answer from a short list of alternatives that are preselected by the examiner. The MCQ examination format is most frequently used in medical education due to its convenience for testing and grading large-size classes [2, 3]. Various experts discourage the use of MCQs, arguing that they promote memorization and factual recall, and that they do not encourage or test high-level cognitive processes such as reasoning or problem solving [4, 5]. As an alternative to multiple-choice examinations, EMQs have been developed to test a student’s knowledge in a more applied and in-depth sense [6–10]. During an extended matching test, the student selects the best answer from a list of 9–26 options, each of which may be used once, more than once, or not at all. Extended matching tests have been reported to be more reliable, better able to monitor progress during a course, associated with a reduced opportunity for students to ‘guess’ the correct answer, and well suited to test core knowledge and clinical reasoning in students compared with the MCQ examination format [7, 11–14].

In the context of the binding study advice and ongoing efforts to improve the quality of medical examination, we aimed to determine the capacity of MCQ, EMQ, and mixed examinations to distinguish between poor (grade <6), moderate (grade 6–8) and excellent students (grade ≥8). We further explored whether sex, age and examination preference were related to MCQ and EMQ examination scores.

## Methods

### Participants

The present study was performed among 452 first-year students studying Medicine (*n* = 351) and Biomedical Sciences (*n* = 101) at the Radboud University Nijmegen, the Netherlands, in the 2011–2012 academic year. Examination scores and responses to questionnaires were linked to each student’s identification number in order not to disclose the identity of the student. Students were informed about the study and their consent was obtained. Ethical approval was waived for this study. Nevertheless, the ethical principles of the Declaration of Helsinki were taken into account during the study design, data collection and data analysis phases.

### Medicine and biomedical science study programmes

Interested students were eligible to apply for admission to the Medicine or Biomedical Science study programmes if they had obtained a diploma in pre-university education (e.g. VWO, Athenaeum or Gymnasium) with courses in biology, chemistry, physics and mathematics. Due to a *numerus fixus*, only a limited number of students can be accepted annually. Eligible students were accepted into the Biomedical Science programme using a lottery system based on average high school grades, with higher grades indicative of a greater chance for acceptance to the programme. For the Medicine programme, 50 % of the available positions were allocated using a similar lottery system. The other 50 % were allocated via a selection procedure in which eligible students completed a matriculation exam. Subsequently, examination scores were ranked and a top–down procedure was followed to allocate the remaining 50 % of available positions [15]. Both study programmes include a 3-year Bachelor’s phase (i.e. undergraduate), followed by a 2- or 3-year Master’s phase (i.e. graduate) for Biomedical Sciences and Medicine, respectively. During the first year of both study programmes, the majority of the courses (60 %) are taken together.

### Procedures

Scores were collected during three different courses in the propaedeutic year. In chronological order, students first completed an EMQ examination (course: Principles of functional morphology), followed by an MCQ examination (course: Biochemical and physical processes), and finally a mixed EMQ and MCQ examination (course: Circulation and respiration). Grades can vary between 0 (lowest score) and 10 (highest score). Students pass a course if they obtain a grade ≥6. Students who obtain a score ≥8 are considered to be excellent.

All students were asked to complete a structured questionnaire related to sex, age, and preferences for examination format. Students were also requested to sign informed consent for participation in this study. All forms were checked for completeness by the observers who were present during the examination. The final grades per course were obtained from the Department for Evaluation, Quality and Development of Medical Education of the Radboud University Nijmegen Medical Centre. For the mixed examination, the overall score as well as the EMQ and MCQ sub-score were included for further analysis.

### Examination formats

#### Multiple choice questions

Multiple-choice evaluation required students to choose the correct answer from a short list of possible answers: 3–5 alternatives that were preselected by the examiner [16]. Correction for guessing was applied to prevent random guessing by the students and thereby obtaining higher grades [17].

#### Extended matching questions

EMQs are problem-focused questions often referring to realistic cases [6]. They have four components: (i) a theme, (ii) a lead-in statement for the questions giving the students instructions on what to do, (iii) the questions giving students pertinent information based on which the student is to select the correct answer and (iv) a list of options or answer possibilities. In the EMQs, students were asked to select the best answer from a list of 9–26 options that were preselected by the examiner, each of which could be used once, more than once, or not at all.

#### Mixed examination

The mixed examination combined EMQ and MCQ questions to test the knowledge of the students. The characteristics of both types of questions were in agreement with the EMQ only and MCQ only examinations, as described above.

### Statistical analyses

Statistical analyses were performed using the Statistical Package for the Social Sciences (IBM SPSS Statistics for Windows, Version 20.0. Armonk, NY: IBM Corp.). Quantitative data were summarized by mean and standard deviation (SD); categorical variables were presented by percentage. The difference in examination score between the MCQ, EMQ, and mixed examination was assessed using one-way analysis of variance (ANOVA). Subsequently, the average examination score over the three tests was calculated, and two new categorical variables were created. First a dummy variable was introduced to distinguish ‘poor’ (average examination score <6) from ‘moderate’ students (average examination score ≥6). The second dummy variable distinguished between ‘excellent’ and ‘other’ students (average examination score ≥8 versus <8, respectively). Using binary logistic regression analysis, we were able to determine the discriminative capacity of MCQ, EMQ and mixed examinations to detect poor (score <6) or excellent (score ≥8) students. The MCQ examination was used as the reference format in both analyses. Risk ratios (RR) were presented with their 95 % confidence intervals (CI). Finally, we created another new variable to compare the characteristics between poor, moderate and excellent students (average examination score <6, 6–8, or ≥8, respectively). Differences between the three groups of students were assessed using one-way ANOVA (continuous parameters) or Pearson’s *χ* ^2^ tests (nominal parameters). Significance was declared if *p* ≤ 0.05.

## Results

### Participants

A total of 413 out of 452 participants completed the questionnaire. As 12 students did not provide informed consent, a total of 401 students were included in the data analysis. Mean age of the respondents was 18.8 years (SD 1.1) and ranged from 17 to 25 years. Participants were predominantly female (female: 65.4 %, male: 34.6 %). Most students had a preference for MCQs as examination format, i.e. 42.8 %, followed by EMQs (29 %), and no preference (28.2 %).

### Examination scores

Mean scores differed (*p* < 0.001) between the MCQ, EMQ and mixed examinations, with grades of 6.8 (SD 1.5), 7.4 (SD 1.3) and 6.5 (SD 1.0), respectively (Table 1). The average examination score over the three different examinations was 6.9 (SD 1.1). Using the average examination grade, 91 students were classified as poor (19 %), 290 students as moderate (61 %), and 98 students as excellent (21 %).

**Table 1:** Average grades and number of students per examination type

	Type of exam		
	MCQ	EMQ	Mixed
Average examination score	6.8 ± 1.5	7.4 ± 1.3	6.5 ± 1.0
Student groups			
Score <6 (%)	21	8	21
Score 6–8 (%)	45	42	67
Score ≥8 (%)	34	50	12

### Type of examination

Logistic regression analysis revealed that EMQ examination was less powerful (RR 0.33, CI 0.22–0.49) to discriminate poor students compared with MCQ examination, while the mixed examination had a comparable discriminative value (RR 0.98, CI 0.71–1.37). In contrast, EMQ examination was more powerful (RR 1.93, CI 1.47–2.53) to identify excellent students compared with MCQ examination, while the mixed examination had a significantly lower discriminative capacity (RR 0.26, CI 0.18–0.37). We also calculated the relative contribution (ratio) of the score that the student obtained in MCQs and EMQs during the mixed examination (Table 2). While poor students predominantly benefit from EMQ compared with MCQ questions (60 versus 40 % of the score), this is perfectly balanced in excellent students (50 versus 50 %).

**Table 2:** Overview of total and categorized score of the *mixed examination*

Total score	*N*	EMQ core	Ratio EMQ: total	MCQs score	Ratio MCQ: total
≤5	22	5.2 (0.89)	0.60:1	3.4 (0.88)	0.40:1
5–5.5	68	6.2 (0.68)	0.59:1	4.4 (0.79)	0.41:1
6–6.5	164	7.0 (0.60)	0.55:1	5.6 (0.76)	0.45:1
7–7.5	127	7.7 (0.61)	0.53:1	6.7 (0.61)	0.47:1
8–8.5	47	8.2 (0.42)	0.51:1	7.9 (0.52)	0.49:1
≥9	3	9.2 (0.16)	0.50:1	9.2 (0.18)	0.50:1

*EMQs* and* MCQs* are presented as mean scores (standard deviation)

*EMQs* extended matching questions, *MCQs* multiple choice questions

### Sex, age and examination preference

**Fig. 1:**
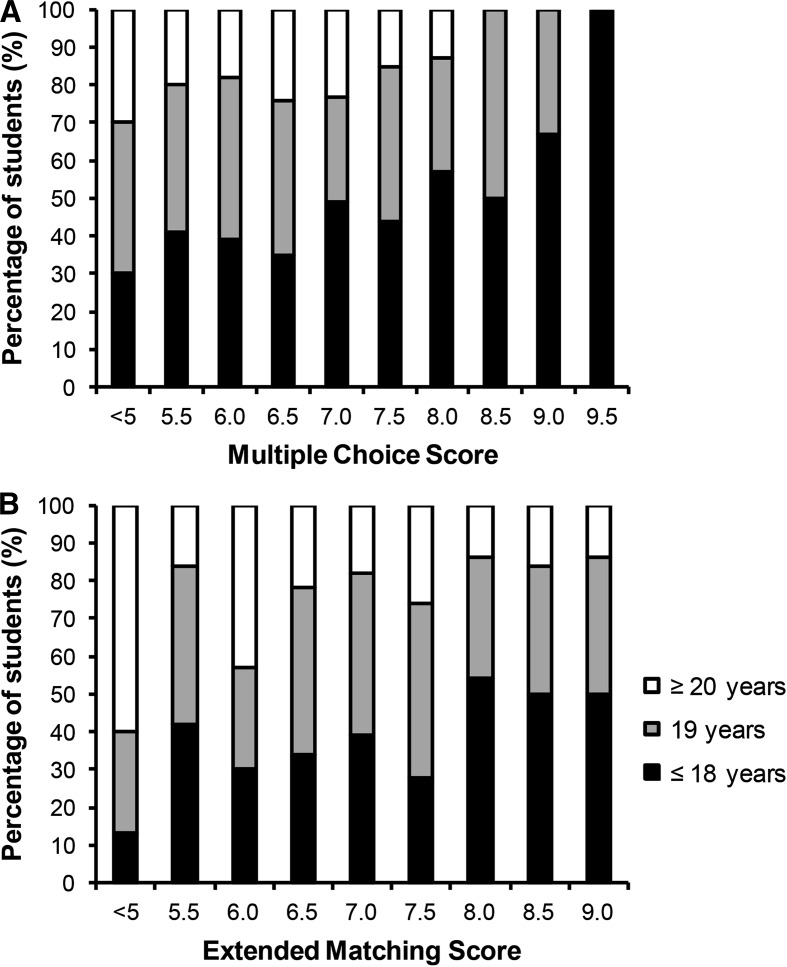
Age classification per grade for **a** multiple-choice questions and **b** extended matching questions in the mixed examination course

To obtain more insight into factors that contribute to the qualification of the student, the characteristics of poor, moderate and excellent students are presented in Table 3. Poor students were older than moderate (*p* = 0.001) and excellent students (*p* < 0.001), while no differences were detected in sex or examination preferences across groups. Age was subsequently classified as ≤18 years (*n* = 159), 19 years (*n* = 154) or ≥20 years (*n* = 87). Students ≥20 years had lower grades compared with the 19- and ≤18-year-old groups for EMQ (6.8 ± 1.1, 7.2 ± 0.9 and 7.3 ± 0.9, respectively; *p* < 0.05) and MCQ (5.5 ± 1.4, 5.8 ± 1.4 and 6.2 ± 1.3, respectively; *p* < 0.05) during the mixed examination course. Figure 1 shows the impact of age category on EMQ and MCQ scores. In addition, the number of poor students differed between students in the ≤18 year (11 %), 19 year (15 %) and ≥20 year (33 %) category (*p* < 0.001). Overall, students ≥20 years had a 4.1 times higher risk to obtain an average grade <6 compared with students ≤18 years (RR 4.1, CI 2.1–8.0).

**Table 3:** Characteristics of poor (<6), average (6–8) and excellent (≥8) students

	Average examination score			
	<6	6–8	≥8	*p* value
Sex				0.35
Men (%)	36	32	41	
Women (%)	64	68	59	
Age (years)	19.4 ± 1.5	18.8 ± 1.1	18.6 ± 0.8	<0.001
Examination preference				0.37
MCQ (%)	52	40	43	
EMQ (%)	28	29	30	
No preference (%)	20	31	26	
Average examination score	5.2 ± 0.5	6.9 ± 0.6	8.4 ± 0.3	<0.001
MCQ exam score	4.8 ± 1.2	6.9 ± 0.9	8.6 ± 0.6	<0.001
EMQ exam score	5.8 ± 1.1	7.4 ± 0.9	8.7 ± 0.4	<0.001
Mixed exam score				
Total score	5.3 ± 0.8	6.5 ± 0.7	7.7 ± 0.6	<0.001
MCQ score	4.3 ± 1.1	5.9 ± 1.0	7.5 ± 0.9	<0.001
EMQ score	6.2 ± 1.0	7.1 ± 0.8	8.0 ± 0.7	<0.001

Values are presented as mean ± SD

## Discussion

In recent years, medical faculties in the Netherlands have implemented a legally binding study advice for all enrolled students in Bachelor programmes [1, 18–24]. This implementation will allow educational organizations to provide better study guidance to students, assuring a higher outcome of students finalizing their study programme and, thereby, increasing effectiveness. Accordingly, appropriate examination and format styles are required to make meaningful distinctions between students at different knowledge levels, leading to valid pass or fail decisions. This study compared the discriminative capacity of EMQ and MCQ for identifying poor (grade <6) and excellent (grade ≥8) students. We further explored the relation between sex, age and examination preference and the examination scores.

In contrast to our hypothesis, we found that MCQ and a mixed examination format including MCQs were the best examination tools to distinguish poor from moderate and excellent students. Both examination formats could identify students with a poor average examination grade (<6) three times better compared with EMQ. Thus MCQs are superior to EMQs in detecting poor students. These findings are in contrast with a previous study that indicated that uncued and extended matching tests have the highest discrimination scores, followed by middle scores for multiple-choice tests, and the lowest discrimination scores for true/false questions [11].

A potential explanation for our discrepant results may relate to the processing of the examinations: MCQs were corrected for guessing while EMQs were not. This may affect the scores in two ways. First, studies have demonstrated that students will guess the most likely answer option if there is no correction for guessing applied to the examination [25]. Accordingly, the examination grade may not be an accurate reflection of their capacity and knowledge, because students can achieve artificially inflated scores through guessing [17, 26, 27]. Although the chance of guessing the right answer in EMQ is low and an elevated cut-off score for EMQ examinations (65 % of highest obtained score) can partially correct for the higher scores, this may have influenced the EMQ grades. Indeed, only 8 % of the students failed the EMQ examination, which is significantly lower compared with the MCQ (21 %) and mixed examination (21 %). The uncorrected EMQs, therefore, seem to disqualify the identification of poor students. Future studies should reveal whether EMQ examinations corrected for guessing can identify both excellent as well as poor students to an equal extent.

The application of correction for guessing in MCQ examinations may also impact the scores of students. As a portion of a mark is deducted when a wrong answer is given, students can choose to leave questions unanswered. Some authors suggest that the application of correction for guessing on MCQ examinations may test risk-taking behaviour rather than the subject-specific knowledge of students [27–29], and introducing a penalty for wrong answers can also help to distinguish between poor- and well-performing students. Our study confirms the latter hypothesis as MCQ was the best examination strategy to distinguish poor from moderate and excellent students. Due to the negative marking of correction for guessing at MCQ, non-learning and poor students may avoid guessing, resulting in a superior discriminative capacity of MCQ compared with EMQ.

We explored the effects of sex, age and examination preference on the examination scores. Neither sex nor examination preference had an influence on the average grade of the students. Interestingly, we did find an inverse relationship between age and examination score. Current evidence is conflicting regarding the role of age on student performance. While some studies report a positive effect of maturing on performance [30, 31], others report a negative effect [32]. We demonstrated significantly lower grades for older students in both EMQ and MCQ examinations, with a substantially higher risk to obtain an average grade <6, which might lead to a negative binding study advice. This finding may relate to the background of the students. Possible explanations could be that older students (1) used more time to finish high school, (2) were excluded multiple times from the study programme due to the *numerus fixus* (e.g. due to low average high school grades), or (3) retook the courses which they failed the year before. Despite a conclusive explanation for our data, we clearly show that students over the age of 20 perform less well compared with their younger peers.

The strengths of this study relate to the large group of students who were included, and the comparison of EMQ and MCQ between and within examinations. However, some limitations should be taken into account. In this study we compared three different examination types during three different courses. Although one might suggest that the content of the course may have influenced the examination scores, we found similar findings regarding the discriminative capacity of EMQ and MCQ across courses as well as within the mixed examination course. Secondly, this study focused on distinguishing poor from moderate and excellent students only, as this information provides an evidence-based and optimal examination strategy that supports the binding study advice. We acknowledge that other examination strategies may be more valuable in identifying excellent students for extra-curricular training. The use of MCQs should therefore only be applied if it serves the primary goal of the examination.

## Conclusion

In the light of the recent implementation of the binding study advice, this study provides relevant insights into the type of examination format with the best discriminative capacity. MCQ is preferred to EMQ with respect to the identification of students with an average grade <6. As MCQ exams have the potential to assess a broad array of topics in a single examination with relatively little grading effort in contrast to open answer questions [26, 33, 34], this type of examination format provides additional benefits for study programmes with large cohorts (e.g. medicine and biomedical sciences). We have also shown that EMQ is superior in identifying excellent students, and future studies should indicate if the application of correction for guessing in EMQ examinations can improve the discriminative capacity of poor students too. Finally, we demonstrated that ‘older’ students perform less well compared with their younger counterparts, while sex and examination preference did not impact the score. These statistics can be taken into account while the binding study advice committee makes its decisions.

## Essentials

- Multiple-choice question examinations possessed a three times better discriminative capacity to identify poorly performing students (average grade ≤6) compared with extended-matching question examinations. To effectively distinguish between poor and moderate/good students for the binding study advice, implementation of multiple-choice question examinations is recommended in the propaedeutic phase of medicine and biomedical science study programmes.
- Extended-matching question examinations better identified students with an average grade ≥8 (excellent) compared with multiple-choice question examinations.
- Correction for guessing is thought to have a major impact on the discriminative capacity of extended-matching and multiple-choice question examinations to identify students with an average grade <6
- Students ≥20 years had a four times higher risk to obtain an average grade <6 compared with students ≤18 years.
- Age and examination preference did not impact the scores for extended-matching and multiple-choice question examinations.
